# Falls, Frailty and Quality of Life Among Individuals on a Regular Haemodialysis Programme: Implications for Rehabilitation Nursing

**DOI:** 10.3390/ijerph23010015

**Published:** 2025-12-20

**Authors:** Marisa Patrícia de Almeida Martins, Ana da Conceição Alves Faria, Carla Gomes da Rocha, Elaine Forte, Letícia de Lima Trindade, Samuel Spiegelberg Zuge, Maria Narcisa da Costa Gonçalves, Olga Maria Pimenta Lopes Ribeiro

**Affiliations:** 1 Porto Hemodialysis Unit, 4200-227 Porto, Portugal; marisa.martins@freseniusmedicalcare.com; 2 Department of Nursing, Santa Maria Health School, 4049-024 Porto, Portugal; ana.faria@santamariasaude.pt; 3 Department of Nursing, School of Health Sciences, HES-SO Valais-Wallis, 1950 Sion, Switzerland; carla.gomesdarocha@hevs.ch; 4 Department of Nursing, Federal University of Santa Catarina, Florianópolis 88053-750, Santa Catarina, Brazil; elaine.forte@ufsc.br; 5 Department of Nursing, Regional Community University of Chapecó (Unochapecó), Chapecó 89809-900, Santa Catarina, Brazil; leticia.trindade@unochapeco.edu.br (L.d.L.T.); samuel.zuge@unochapeco.edu.br (S.S.Z.); 6 Department of Nursing, Nursing School of Porto, 4200-072 Porto, Portugal; mnarcisa@esenf.pt; 7 RISE-Health, 4200-319 Porto, Portugal

**Keywords:** haemodialysis, falls, frailty, quality of life, rehabilitation nursing

## Abstract

Background: Chronic kidney disease and haemodialysis treatment are associated with physiological and functional alterations that compromise postural stability, favouring frailty and the risk of falls. These conditions directly affect the quality of life and autonomy of people undergoing haemodialysis, constituting an important challenge for rehabilitation nursing. In this sense, the aim of this study was to analyse the relationship between falls, frailty and quality of life in people with chronic kidney disease on a regular haemodialysis programme, identifying implications for rehabilitation nursing care. Methods: This was a quantitative, observational and cross-sectional study conducted with 62 participants from a haemodialysis unit in northern Portugal. The Tilburg Frailty Indicator and the Kidney Disease Quality of Life Instrument (KDQOL-SF™ 1.3) were applied. Statistical analysis used parametric and non-parametric tests, considering a significance level of *p* < 0.05. Results: The prevalence of falls in the year preceding the data collection was 32.2%, and the prevalence of frailty was 40.3%. A significant association was found between frailty and falls (*p* = 0.038) and between sex and falls (*p* = 0.002). The dimensions Symptoms/problems and Effects of kidney disease on daily life showed lower scores among participants with falls (*p* < 0.001). Conclusions: Frailty and poorer illness perception were associated with the occurrence of falls and with lower quality of life. Comprehensive assessment and the implementation of rehabilitation programmes led by specialist nurses in rehabilitation nursing are essential to promote functionality, safety and autonomy in people undergoing haemodialysis.

## 1. Introduction

Chronic Kidney Disease (CKD) is a progressive and irreversible condition, characterised by the gradual loss of kidney function and by a combination of metabolic, haemodynamic and musculoskeletal alterations that significantly compromise the functionality and quality of life of the affected individuals [1,2]. It is a complex and highly prevalent pathology, whose incidence has been increasing with population ageing and the prevalence of chronic diseases such as arterial hypertension and diabetes mellitus, representing an important challenge for contemporary health systems.

In the terminal stages of the disease, Haemodialysis (HD) often emerges as the most appropriate renal replacement therapy, allowing the maintenance of life and the control of uraemic symptoms [3,4]. However, this therapeutic modality entails a considerable physical, emotional and social burden. During the dialysis process, complications such as hypotension, nausea, headaches, cramps and chest or lower back pain may occur [2,4,5]. These manifestations, resulting from haemodynamic variations and the disruption of homeostasis, have repercussions on body balance and postural stability, particularly in the post-dialysis period, during which greater vulnerability to adverse events is observed [6,7].

The literature has shown that individuals with CKD undergoing HD present clinical conditions with a high systemic impact, associated with gradual declines in strength, endurance, gait speed and energy reserve, with direct consequences for functionality, self-care and autonomy [8,9,10]. These impairments fall within the conceptual spectrum of frailty, understood as a multidimensional syndrome that reflects a reduction in the organism’s adaptive capacity and an increase in vulnerability to physiological and psychosocial stressors. Thus, frailty results in an increased risk of disability, hospitalisation, dependency and mortality [11].

The prevalence of frailty in people undergoing HD is about five times higher than that observed in the healthy population [12], reaching values between 30% and 45% [9]. Although frequently associated with progressive functional deterioration, manifested by postural instability, slow gait, reduced muscle strength and exhaustion, it is a potentially reversible condition and, therefore, a priority domain of attention in Rehabilitation Nursing [11,13,14].

Frailty and the functional deterioration associated with CKD and HD have direct implications for the increased risk of falls. It is estimated that around 25% of people on a regular HD programme experience at least one fall per year, with an incidence higher than that of the general population [15]. Falls occur more frequently in the post-dialysis period, when factors such as hypotension, muscle fatigue, imbalance, weakness, and proprioceptive and cognitive alterations favour the occurrence of fractures, injuries and functional decline [16,17]. In addition to the physical consequences, falls have psychological and social impacts, generating activity limitation and isolation, which further accentuate the cycle of inactivity and dependence in self-care.

In this context, the quality of life of people with CKD is affected across multiple dimensions—physical, psychological, social and occupational. Studies indicate that the most impaired dimensions are physical function, physical condition, pain, general health, emotional well-being, social function, energy/fatigue and occupational activity, reflecting the global impact of the disease and of HD treatment [18,19]. Functional limitation, fatigue, dependence on treatment, polypharmacy and falls directly interfere with autonomy and social participation. At the same time, emotional changes, isolation and the loss of social roles enhance psychological distress and a negative perception of health. These factors, combined with frailty, reinforce the importance of person-centred interventions focused on functionality and overall well-being, promoting an integrated approach that encompasses physical, cognitive and emotional dimensions [20,21]. Considering that these aspects fall within the specific competences of Specialist Nurses in Rehabilitation Nursing (SNRN) [13,22], it becomes essential to create conditions that allow people on regular HD programmes to benefit from interventions that, in addition to preventing the onset and/or worsening of frailty and its consequent complications, simultaneously ensure better quality of life.

Studies show that rehabilitation programmes incorporating therapeutic exercise, balance training, energy conservation strategies, optimisation of self-care, and education for chronic condition management contribute to improvements in muscle strength, functional capacity, balance, and mobility, while simultaneously reducing functional decline and the risk of falls. Accordingly, specialist nurses in rehabilitation nursing play a central role in the care of these individuals, given their ability to implement individualised interventions focused on functionality, safety, and autonomy, thereby constituting a key element in the prevention and management of frailty in the haemodialysis context [6,11,15].

Understanding how the changes resulting from CKD and HD treatment relate to the occurrence of falls, the condition of frailty and quality of life is essential for outlining intervention strategies that support prevention, rehabilitation and functional readaptation of people on regular HD programmes. Such knowledge contributes to adaptation to the chronic condition, mitigation of complications and adverse events, participation in self-care and improvement of quality of life [23,24,25].

In this sense, the aim of the present study was to analyse the relationship between falls, frailty and quality of life in people on a regular HD programme, with a view to identifying implications and contributions for the development of Rehabilitation Nursing programmes that promote functionality, adaptation to the chronic condition, safety and autonomy among people with CKD.

## 2. Materials and Methods

### 2.1. Study Design and Participants

This was a quantitative, observational study, presented with support from the Strengthening the Reporting of Observational Studies in Epidemiology (STROBE^®^) tool [26]. The target population of this study consisted of people on a regular HD programme in a HD unit belonging to a hospital institution located in northern Portugal. A non-probabilistic convenience sampling technique was used to select participants. The inclusion criteria were defined as follows: people aged 18 years or older, diagnosed with chronic kidney disease, and undergoing a regular HD programme for at least three months. The exclusion criteria were: people undergoing other types of renal replacement therapy, as well as those with communication impairments, significant hearing deficits, or cognitive impairments that prevented understanding of the questions. Cognitive deficit was assessed using the Mini-Mental State Examination. Based on the defined criteria, from a population of 100 patients, a sample of 62 participants was obtained. Twenty patients were excluded for not meeting the eligibility criteria, while 18 declined to participate in the study.

### 2.2. Instruments and Variables

As a data collection instrument, a form consisting of five parts was used: Part I—Sociodemographic characteristics of the participant; Part II—Health condition of the participant; Part III—Characterisation of falls; Part IV—Frailty; and Part V—Quality of Life.

Regarding sociodemographic and professional variables, the following were explored: sex, age, marital status, level of education, occupation and income. With respect to the health condition, the variables included personal medical history, signs and symptoms, regular medication, time since diagnosis of kidney disease, time on the HD programme and the need for walking aids. For the characterisation of falls, data were collected regarding their occurrence, location and associated consequences. The questions used to characterise falls were developed by the authors.

With respect to frailty, the Tilburg Frailty Indicator (TFI) was used, adapted and validated for the Portuguese population by Coelho (2014), which enables the assessment of the physical, psychological, and social domains [27]. The physical domain of the TFI comprises eight items addressing physical health unexplained weight loss, difficulty in walking, difficulty in maintaining balance, hearing problems, vision problems, lack of strength in hands and physical tiredness. The psychological domain includes four items related to cognitive function, depressive/anxiety symptoms, and coping mechanisms. The social domain consists of three items: living alone, social relationships, and social support. TFI internal consistency was good (Kuder-Richardson formula (KR-20) = 0.78) [27]. Regarding the final frailty score, individuals with scores equal to or greater than 6 are considered frail, with higher scores indicating greater frailty.

To assess the quality of life of people with renal insufficiency on a HD programme, the Kidney Disease Quality of Life Instrument (KDQOL-SF™ version 1.3) was used, adapted and validated for the Portuguese population by Ferreira & Anes (2010) [28]. This instrument includes specific items distributed across eight dimensions that focus on the concerns of dialysis people: Symptoms/problems, Effects of kidney disease on daily life, Burden of kidney disease, and the impact on Work status, Cognitive function, Quality of social interaction, Sexual function and Sleep. It also encompasses three quality-of-life dimensions: Social support, Dialysis staff encouragement and Patient satisfaction. The KDQOL-SF™ also includes version 2 of the SF-36, with 36 questions grouped into eight dimensions: Physical functioning, Physical role, Pain, General health, Emotional well-being, Emotional functioning, Social functioning and Energy/fatigue. Internal consistency, or content homogeneity, was assessed using Cronbach’s alpha, with values above 0.70, except for Work status (0.69), Burden of kidney disease (0.65), and Quality of social interaction (0.36) [28]. The maximum score for each dimension is 100, with higher scores indicating better status in that dimension.

### 2.3. Procedure

Data collection was carried out between January and February 2024 through the face-to-face administration of a form by the same researcher, with an average completion time of 40 min. Contact with each participant took place in the HD room during the dialysis procedure.

### 2.4. Statistical Analysis

Statistical analysis was conducted using the Statistical Package for the Social Sciences (SPSS), version 29.0. An item matrix was constructed, preserving the original ordering of the form in order to reduce potential reading errors and facilitate the data transfer process. Initially, descriptive analysis was performed, with calculation of central tendency measures (mean) and dispersion (standard deviation), as well as the determination of proportions expressed as percentages. In a second stage, inferential analysis was carried out to identify relationships between the variables. A significance level of 5% (*p* < 0.05) was adopted. Parametric and non-parametric tests were used according to the nature and distribution of the variables. Among the parametric tests, Student’s *t*-test and the Shapiro–Wilk test were used. The non-parametric tests included the Lilliefors (Kolmogorov–Smirnov) test and the Chi-square test (for quantitative variables), as well as the Mann–Whitney test (for qualitative variables). The odds ratio measure was used to assess the likelihood of falls occurring between different groups.

The normality tests—Shapiro–Wilk and Lilliefors (Kolmogorov–Smirnov)—were applied to verify whether the samples originated from populations with a normal distribution, thereby determining the subsequent statistical procedures. In this context, the Chi-square test was used to test the independence between qualitative variables. Rejection of this hypothesis led to the conclusion that an interaction existed between the two variables. When the sample size was small, Fisher’s exact test was used.

The Cochran–Armitage test was used to test the hypothesis of an association between a nominal variable with two categories (the occurrence of falls) and an ordinal variable with several categories (such as the dimensions of frailty). Student’s *t*-test was used to test the equality of means (equal location) of two normal populations (independent samples). It was applied when normality for both variables was not rejected. The Mann–Whitney test was used to test the same hypothesis as Student’s *t*-test, but when the variables involved did not follow a normal distribution.

### 2.5. Ethical Considerations

The study received approval from the Ethics Committee (310/2023) and authorisation from the Board of Directors of the hospital institution in northern Portugal. Be-fore acceptance, the aims of the study were explained to potential participants, ensuring their right to refuse or withdraw at any stage of the process if they felt uncomfortable for any reason. Patients who agreed to participate provided informed consent prior to the administration of the form. It was also emphasised that the responses were anonymous and confidential. Only the research team had access to the collected data.

## 3. Results

The results are divided into six topics: sociodemographic characterisation, health condition, characterisation of falls, frailty status, quality of life and comparisons between the variables under study and the occurrence of falls.

### 3.1. Sociodemographic Characterisation of the Participants

The individuals who participated in the study were predominantly male (n = 38; 61.3%). The mean age of the participants was 69.97 ± 11.69 years, with ages ranging from 40 to 92 years. Regarding marital status, 61.3% (n = 38) were married or living in a common-law partnership, and the lowest percentage corresponded to single participants (n = 5; 8.1%).

Educational attainment was predominantly low, with the highest percentage, 61.3% (n = 38), corresponding to primary education, and only 4.8% (n = 3) having completed higher education. Analysis of the collected data showed that 83.9% of the participants (n = 52) were already retired. Regarding participants’ income, the majority (62.9%, n = 39) reported earning less than 750 euros, 30.6% (n = 19) reported an income between 750 and 2000 euros, and only 6.5% (n = 4) reported an income above 2000 euros (Table 1).

### 3.2. Characterisation of the Health Status of the Participants

The following data refer to the health status of the participants (Table 2). Regarding personal medical history, cardiovascular disease was predominant (n = 26; 41.9%), followed by metabolic disease (n = 18; 29.0%). With respect to signs and symptoms, reported by 35 participants (56.5%), the most frequent were dizziness and imbalance, each reported by 11 participants (31.4%), while the least frequent were weight loss and foot pain, each reported by one patient (2.9%). Medication use was marked particularly by antihypertensive drugs (n = 29; 46.8%) and antidiabetic drugs (n = 16; 25.8%). The mean time since diagnosis of kidney disease was 10.62 ± 9.64 years, and the time on HD was 3.97 ± 3.82 years. Of the 62 participants (100%), 19 (30.6%) used walking aids. It was found that the most commonly used aid, according to the participants, was the cane (n = 13; 68.4%).

### 3.3. Characterisation of Participants’ Falls

In the year preceding the data collection, 20 participants (32.2%) had a history of falls, with a total of 35 falls reported. Among these, 16 participants (80%) experienced only one episode. Of the 35 falls reported, most occurred at home (n = 19; 54.3%) and after dialysis (n = 17; 48.6%). The most frequent type was a fall from standing height (n = 28; 80%), and most resulted in no injuries (n = 20; 57.1%) (Table 3).

### 3.4. Characterisation of Participants’ Frailty Status

According to the assessment instrument used and considering its defined criteria—that is, a frailty score equal to or greater than six—25 participants (40.3%) were found to present a condition of frailty (Table 4).

### 3.5. Characterisation of Participants’ Quality of Life

Regarding quality of life, according to the assessment instrument, the dimensions with the highest scores were Dialysis staff encouragement (Mean = 96.98), Quality of social interaction (Mean = 79.25) and Cognitive function (Mean = 79.03). The dimensions in which participants showed the lowest quality of life were Work status (Mean = 11.29), Burden of kidney disease (Mean = 29.13), Physical functioning (Mean = 42.34) and General health (Mean = 44.11) (Table 5).

### 3.6. Comparisons Between the Variables Under Study and the Occurrence of Falls

When analysing sex and the occurrence of falls, the Chi-square test was found to be significant (*p* = 0.002), leading to the conclusion that women are more likely to experience falls than men.

When analysing marital status and the occurrence of falls, only married individuals and a combined category of “others” (including widowed, single and divorced participants) were considered for the Chi-square test, due to the small number of individuals in the latter categories. It was found that the Chi-square test was not significant (*p* = 0.124). Therefore, it was concluded that there is no statistically significant difference in the occurrence of falls between married participants and those in other marital status categories.

Before analysing age and the occurrence of falls, it was verified that the Shapiro–Wilk and Lilliefors (Kolmogorov–Smirnov) normality tests rejected the hypothesis of normal age distribution (*p* = 0.016 and *p* = 0.003, respectively). Therefore, this comparison was performed using the Mann–Whitney test, whose result was not statistically significant (*p* = 0.456), leading to the conclusion that there is no difference in the mean age of individuals with and without falls. Thus, in this study, no relationship was found between age and the occurrence of falls. In fact, the mean age of individuals with and without falls was 72 years and 69 years, respectively.

Regarding education level and the occurrence of falls, it was not possible to perform the comparison, as almost all individuals with falls had only primary education. In fact, only three individuals with falls had other levels of education.

With respect to signs and symptoms and the occurrence of falls, it was only possible to distinguish dizziness, grouping all other signs/symptoms together due to the small number of observations in some categories. For the same reason, the comparison was performed using Fisher’s exact test. The result was not significant (*p* = 0.412), leading to the conclusion that there is no statistically significant difference in the occurrence of falls between participants with dizziness and those with other signs/symptoms.

To analyse frailty status and the occurrence of falls, the Mann–Whitney test was used. Regarding overall frailty, the result was statistically significant (*p* = 0.038), leading to the conclusion that the mean overall frailty score of individuals who experienced falls is higher than that of those who did not.

Regarding quality of life and the occurrence of falls, Table 6 presents the results of the comparison tests applied to the various dimensions of the quality-of-life scale between individuals with and without falls. Initially, normality in the dimensions was assessed using the Shapiro–Wilk and Lilliefors (Kolmogorov–Smirnov) tests. The comparison was performed using the Mann–Whitney test when normality was rejected by at least one of the tests, and using Student’s *t*-test otherwise.

Concerning the dimensions Work status, Sexual function, Dialysis staff encouragement, Emotional well-being and Energy/fatigue, due to the small number of individuals in some categories, it was not possible to perform any statistical test.

In the Symptoms/problems dimension, the tests rejected normality for individuals with falls (*p* < 0.001 in both tests) and for those without falls (*p* = 0.009 and *p* = 0.017, respectively). The result of the Mann–Whitney test was statistically significant (*p* < 0.001), leading to the conclusion that the mean score for this dimension among individuals with falls is lower than that of those without falls.

In the Effects of kidney disease on daily life dimension, the tests rejected normality for individuals with falls (*p* < 0.001 in both tests). For individuals without falls, the Shapiro–Wilk test rejected normality (*p* = 0.001), whereas the Lilliefors test did not (*p* = 0.056). The result of the Mann–Whitney test was significant (*p* = 0.0004), leading to the conclusion that the mean score for this dimension among individuals with falls is lower than that of those without falls.

## 4. Discussion

The results of this study made it possible to outline a sociodemographic, clinical and functional profile of the participants, as well as to identify associations between variables of interest and the occurrence of falls among individuals undergoing HD.

The sample was predominantly male (61.3%), with a mean age of approximately 70 years, which is consistent with the literature indicating a predominance of older men among patients with CKD undergoing dialysis therapy [10]. The low level of education observed (61.3% with only primary schooling) and the high number of retired individuals (83.9%) reflect a vulnerable socioeconomic profile, a characteristic frequently associated with poorer health outcomes and greater physical and social vulnerability. Most participants had a monthly income below 750 euros, which may have implications for treatment adherence and quality of life.

The presence of comorbidities was high, with a predominance of cardiovascular diseases (41.9%) and metabolic diseases (29.0%), consistent with the typical profile of patients with CKD. Dizziness and imbalance were the most frequently reported symptoms, each by 31.4% of the participants, and these symptoms are closely associated with the risk of falls. The use of multiple medications, particularly antihypertensive and antidiabetic drugs, reinforces the presence of polypharmacy, a factor widely recognised in the literature as contributing to the risk of postural instability and falls [29,30,31].

The incidence of falls in the year preceding the data collection was high (32.2%) and similar to that reported in studies involving dialysis populations, which indicate values close to 32.0% and an average of 1.3 falls per person per year, reinforcing that falls represent a relevant complication among individuals with CKD undergoing regular HD [17,20]. Most falls occurred at home (54.3%) and after the HD session (48.6%), suggesting the direct influence of the treatment on postural instability. During and after HD, haemodynamic alterations, fatigue, intradialytic hypotension and changes in proprioception impair balance and increase vulnerability to falls [11,32]. Although more than half of the falls did not result in injuries (57.1%), the presence of head trauma and fractures in some cases indicates the potential severity of the event and the need for preventive measures. Moreover, even when falls do not result in physical injury, the psychological and functional impact is significant, as fear of falling and loss of confidence may reduce physical activity, favour social isolation and intensify frailty [33].

The prevalence of frailty was high (40.3%), corroborating studies that report prevalence rates between 35% and 45% among CKD patients undergoing dialysis, including individuals under 65 years of age [8,11,34]. This condition is associated with sarcopenia and reduced functional capacity. The statistically significant association between frailty and the occurrence of falls reinforces the evidence that frailty is an important predictor of fall risk in this population. This finding highlights the need for regular screening and intervention programmes aimed at maintaining muscle strength and balance. The literature corroborates that the frailty process tends to worsen with advancing age and the progression of comorbidities, resulting in decreased strength, endurance and reaction time, factors that increase susceptibility to falls [14,35]. Additionally, fatigue, hypotension and autonomic dysfunction associated with dialysis contribute to postural instability and reduced functional capacity [36].

Frailty should be understood as a multidimensional condition, encompassing physical, psychological and social aspects. Fears related to falling, anxiety, depression and a reduced sense of control undermine motor performance and intensify the loss of autonomy. The interaction between frailty and falls is bidirectional: while frailty increases the risk of falling, a fall reinforces the cycle of dependency, fear, isolation and functional decline [37,38].

The assessment of quality of life showed good results in the dimensions related to dialysis staff encouragement and quality of social interaction, reflecting the importance of interpersonal support in the therapeutic context. However, dimensions such as Work status, Burden of kidney disease and Physical functioning presented low scores, indicating significant limitations in physical performance and in the perception of the burden of kidney disease.

In the comparisons between individuals with and without falls, it was observed that the Symptoms/problems and Effects of kidney disease on daily life dimensions showed significantly lower scores among participants with falls. This suggests that worse physical symptoms and a greater impact of the disease on daily life may be associated with increased vulnerability and risk of falls. Although other dimensions did not show statistically significant differences, there was a general tendency toward lower scores among individuals with falls, reflecting the overlap between functional limitations, frailty and reduced quality of life.

Our analysis also showed a significant association between sex and the occurrence of falls, with a higher prevalence among women, a result consistent with previous studies that point to biomechanical differences, hormonal changes and lower bone and muscle density after menopause as predisposing factors for frailty and falls [20]. On the other hand, age did not show a significant association, although the mean age was slightly higher among those who experienced falls, suggesting that more than chronological age, functional and clinical status appear to be the main determinants of fall risk.

Overall, the results indicate that the occurrence of falls among the participants is related not only to physical factors, but also to the presence of frailty and to the perception of poorer quality of life. These findings reinforce the importance of an integrated approach in HD, focused on fall prevention, muscle strength and balance training, management of kidney disease and the promotion of psychosocial support.

In this context, it becomes essential for healthcare teams to carry out periodic assessments of patients’ health status and to identify risk factors, particularly in HD clinics. Many episodes remain underreported, as patients perceive falls as inevitable or fear losing autonomy, which limits early interventions and reinforces the need for educational strategies focused on self-care and safety [33]. The literature highlights the importance of using population-specific instruments, as generic scales such as the Morse Fall Scale and the Hendrich II Fall Risk Assessment Model may not adequately capture the complexity of patients undergoing HD [39].

Structured and supervised physical activity is one of the most effective interventions for preventing falls and reducing frailty. Exercises performed during HD, such as the use of cycle ergometers, promote improvements in cardiovascular function, muscle endurance and quality of life [40]. Intradialytic cycling, when combined with monitoring and individualised feedback, reduces the rate of falls, although multifactorial interventions show a more moderate impact [15,25]. Despite the benefits, adherence to exercise programmes is often limited by psychological, motivational and physical barriers, requiring personalised approaches [41].

Recent studies highlight the need to integrate cognitive and emotional dimensions into rehabilitation, encompassing motor and cardiorespiratory training, activities of daily living, education and psychological support [36]. The American Association of Kidney Patients (AAKP) also recommends home-based preventive measures such as grab bars, non-slip mats, adequate lighting, unobstructed hallways, handrails, appropriate footwear and regular balance and muscle-strengthening exercises [42].

In light of the above, the role of SNRN is highlighted in promoting functionality and autonomy by implementing structured and personalised intervention plans that include motor, cardiorespiratory and activities of daily living training, as well as strategies for disease self-management [13]. The continuous work of the SNRN allows for the early detection of signs of functional decline, enabling preventive action and the promotion of safe and adapted environments [23,25]. It is also relevant to consider psychosocial dimensions, integrating emotional support, encouragement of social participation and empowerment for self-care. Family involvement and multiprofessional coordination are essential for maintaining quality of life and reducing frailty and dependency.

In this way, clinical decision-making, grounded in scientific evidence and in the individual needs of each person, guides the design and implementation of structured programmes that ensure dignity, continuity and quality of care.

Although this is one of the first national studies to examine the relationship between falls, frailty and quality of life among patients undergoing regular HD, it presents limitations inherent to the cross-sectional design and the use of self-reported data, which are susceptible to recall bias, particularly in older individuals. The literature indicates that fall recall tends to underestimate the true prevalence [43,44]. Difficulties in reporting daily medication may also have influenced the results related to polypharmacy [29,30]. The small sample size and the convenience sampling approach limit generalisability, and therefore longitudinal and probabilistic studies are recommended to further analyse the relationship between frailty, falls and rehabilitation nursing interventions in patients undergoing regular HD.

Another limitation of this study relates to the high variability observed in the data. The marked dispersion may have reduced the statistical power of the analyses, hindering the detection of effects or associations that may, in fact, exist. This variability may also reflect greater heterogeneity within the sample, thereby limiting the generalisability of the findings. Accordingly, future studies are recommended to consider strategies aimed at controlling or reducing variability in order to obtain more robust estimates and more precise conclusions.

## 5. Conclusions

The results of this study highlight the high prevalence of falls and frailty among individuals undergoing regular HD, confirming that both conditions are interconnected and associated with poorer quality-of-life indicators. It was observed that frailty, the presence of physical symptoms and the greater impact of kidney disease on daily life are significantly associated with the occurrence of falls, demonstrating that the risk does not depend exclusively on chronological age, but rather on functional, clinical and psychosocial factors. Thus, a comprehensive assessment of the person undergoing HD—encompassing functional status, balance, muscle strength and perceived health—proves essential for prevention and early intervention.

The integration of rehabilitation programmes led by SNRN plays a central role in promoting functionality, autonomy and safety in these patients. Interventions that combine supervised physical training, educational strategies, psychological support and environmental adaptation have proven effective in reducing falls, strengthening confidence and improving quality of life. The implementation of evidence-based practices and collaborative work between healthcare professionals and patients constitute fundamental pillars of person-centred care and of the preservation of dignity and independence.

Considering the limitations of this study—namely the cross-sectional design, the small sample size and the use of self-reported data—longitudinal and multicentre investigations are recommended to provide a deeper understanding of the causal relationships between falls, frailty and quality of life. Future research may also explore the impact of structured rehabilitation nursing programmes across different dialysis settings. In summary, fall prevention and the mitigation of frailty should be recognised as central components of HD care, contributing to safer, more humanised clinical practice and to positive health outcomes.

## Figures and Tables

**Table 1:** Sociodemographic characteristics of the participants.

Sociodemographic Characteristics	Frequency	Percentage
Sex		
Male	38	61.3
Female	24	38.7
Total	62	100
Marital status		
Single	5	8.1
Married/In a common-law partnership	38	61.3
Divorced	7	11.3
Widowed	12	19.4
Total	62	100
Education level		
Illiterate (0 years)	1	1.6
Primary education (1–4 years)	38	61.3
Lower secondary education (5–6 years)	5	8.1
Upper secondary education (7–9 years)	8	12.9
High School	7	11.3
Higher education (Bachelor’s degree or equivalent)	3	4.8
Total	62	100
Occupation		
Unemployed	3	4.8
Retired	52	83.9
Technicians and associate professionals	1	1.6
Administrative staff	1	1.6
Plant and machine operators	1	1.6
Unskilled workers	4	6.5
Total	62	100
Income		
<750 euros	39	62.9
750–2000 euros	19	30.6
>2000 euros	4	6.5
Total	62	100

**Table 2:** Health status of the participants.

Health Status	Frequency	Percentage
Medical History		
Cardiovascular disease	26	41.9
Respiratory disease	6	9.7
Neurological disease	5	8.1
Musculoskeletal disease	4	6.5
Metabolic disease	18	29.0
Oncological disease	3	4.8
Total	62	100
Signs and symptoms		
Dizziness	11	31.4
Imbalance	11	31.4
Muscle weakness	4	11.4
Difficulty walking	7	20.0
Weight loss	1	2.9
Other (foot pain)	1	2.9
Total	35	100
Medication		
Antihypertensive drugs	29	46.8
Antidiabetic drugs	16	25.8
Diuretic drugs	10	16.1
Statin drugs	4	6.5
Anti-inflammatory drugs	3	4.8
Total	62	100
Walking aid		
Crutches	3	15.8
Wheelchair	2	10.5
Walker	1	5.3
Cane	13	68.4
Total	19	100

**Table 3:** Characterisation of falls.

Characteristics of the Fall	Frequency	Percentage
Place of fall		
Transport	1	2.8
Clinic	3	8.6
Home	19	54.3
Street	12	34.3
Total	35	100
Day of occurrence		
Before dialysis	3	8.6
After dialysis	17	48.6
Non-dialysis day	15	42.8
Total	35	100
Height of fall		
From standing height	28	80.0
Above standing height	2	5.7
Below standing height	5	14.3
Total	35	100
Consequences of the fall		
No injuries	20	57.1
Bruise	6	17.1
Dislocation	1	2.9
Traumatic brain injury	2	5.7
Fracture	2	5.7
Incised wound	1	2.9
Hospitalisation	3	8.6
Total	35	100

**Table 4:** Frailty status of the participants.

Frailty Score	Frequency	Percentage
0	5	8.1
1	5	8.1
2	3	4.8
3	11	17.7
4	4	6.5
5	9	14.5
6	6	9.7
7	8	12.9
8	2	3.2
9	6	9.7
10	1	1.6
11	1	1.6
12	1	1.6
Total	62	100

**Table 5:** Quality-of-life dimensions of the participants.

Dimension	Mean	Standard Deviation
Symptoms/problems	78.83	12.07
Effects of kidney disease on daily life	71.78	20.26
Burden of kidney disease	29.13	21.47
Work status	11.29	26.27
Cognitive function	79.03	15.82
Quality of social interaction	79.25	16.33
Sexual function	60.00	10.80
Sleep	65.61	15.81
Social support	75.54	21.70
Dialysis staff encouragement	96.98	10.54
Patient satisfaction	65.05	18.28
Physical functioning	42.34	28.11
Physical role	75.81	35.63
Pain	71.98	25.27
General health	44.11	21.89
Emotional well-being	63.48	15.93
Emotional functioning	77.02	34.86
Social functioning	72.98	23.87
Energy/fatigue	56.45	16.33

**Table 6:** Quality-of-life dimensions among participants with and without falls.

	Falls		Comparison
Dimension	Presence	Absence	*p*
Symptoms/problems	68.2	85.4	<0.001 *
Effects of kidney disease on daily life	57.8	78.4	0.0004 *
Burden of kidney disease	30.3	28.6	0.988 *
Cognitive function	73.7	81.6	0.065 *
Quality of social interaction	76.7	80.5	0.290 *
Sleep	68.5	64.2	0.324 **
Social support	69.2	78.6	0.134 *
Patient satisfaction	67.5	63.9	0.540 *
Physical functioning	35.0	45.8	0.233 *
Pain	73.6	71.2	0.852 *
General health	38.5	46.8	0.182 **
Emotional functioning	57.4	66.4	0.062 *
Social functioning	68.8	75.0	0.247 *

* Mann–Whitney test. ** Student’s *t*-test.

## Data Availability

The data that support the findings of this study are available from the corresponding author upon reasonable request.

## Abbreviations

The following abbreviations are used in this manuscript:

AAKP	American Association of Kidney Patients
CKD	Chronic Kidney Disease
HD	Hemodialysis
SNRN	Specialist Nurses in Rehabilitation Nursing
SPSS	Statistical Package for the Social Sciences

## References

[1] Fletcher B.R., Damery S., Aiyegbusi O.L., Anderson N., Calvert M., Cockwell P., Ferguson J., Horton M., Paap M.C.S., Sidey Gibbons C. (2022). Symptom Burden and Health Related Quality of Life in Chronic Kidney Disease: A Global Systematic Review and Meta-Analysis. PLoS Med..

[2] Wang J., Yao J., Zhu X., Wang T., Lu J., Wei Q., Xue J., Wu Y., You L. (2023). Impact of Frequent Intradialytic Hypotension on Quality of Life in Patients Undergoing Hemodialysis. BMC Nephrol..

[3] İkiz S.N., Usta Y.Y., Sousa C.N., Teles P., Dias V.F.F., Magalhães A.L.P., de Sá Basílio Lins S.M., Ribeiro O.M.P.L. (2019). Validation of the Scale of Assessment of Self-Care Behaviours for Arteriovenous Fistula in Patients Undergoing Haemodialysis in Turkey. J. Ren. Care.

[4] Abdallah A.M., Elsheikh S.M., ElBarbary R.A. (2023). Prevalence and Determinants of Severity of Uremic Pruritus in Hemodialysis Patients: A Multicentric Study. J. Investig. Med..

[5] Singh M., Srinivas Nayak S., Tiwari P., Shaikh S., Gautam V., Waman Ghatol P. (2023). Review Article on Hemodialysis and Its Complications. Asian J. Pharm. Res. Dev..

[6] Pérez-Gurbindo I., Angulo Carrere M.T., Arribas Cobo P., Puerta M., Ortega M., Jaldo M.T., de Sequera P., Alcázar R., Pérez-García R., Álvarez-Méndez A.M. (2020). Haemodialysis Patients Have Worse Postural Balance with an Increase after the Dialysis Session. Nefrologia.

[7] Zhi M., Zeng Y., Chen C., Deng S., Liu Y., Huang Y., Chu B., Hu H. (2025). The Relationship between Intradialytic Hypotension and Health-Related Quality of Life in Patients Undergoing Hemodialysis: A Cross-Sectional. Sci. Rep..

[8] Lee H.-J., Son Y.-J. (2021). Prevalence and Associated Factors of Frailty and Mortality in Patients with End-Stage Renal Disease Undergoing Hemodialysis: A Systematic Review and Meta-Analysis. Int. J. Environ. Res. Public Health.

[9] Kupske J.W., Uggeri F.B., Trindade L.F., Izolan N.F., Keller K.D., Moreira P.R., Krug R.R. (2021). Relação da Fragilidade com Variáveis Clínicas de Pacientes com Insuficiência Renal Crônica. Rev. Recien–Rev. Cient. Enferm..

[10] Sousa C.N., Teles P., Paquete A.R.C., Dias V.F.F., Manzini C.S.S., Nicole A.G., Ozen N. (2022). Effects of Demographic and Clinical Characteristics on Differences in Self-Care Behavior Levels with Arteriovenous Fistula by Hemodialysis Patients: An Ordinal Logistic Regression Approach. Ther. Apher. Dial..

[11] Kennard A., Glasgow N., Rainsford S., Talaulikar G. (2023). Frailty in Chronic Kidney Disease: Challenges in Nephrology Practice. A Rev. Curr. Lit. Intern. Med. J..

[12] Gesualdo G.D., Duarte J.G., Zazzetta M.S., Kusumota L., Orlandi F.S. (2020). Frailty and Associated Risk Factors in Patients with Chronic Kidney Disease on Dialysis. Ciênc. Saúde Coletiva.

[13] Ministério da Saúde (2019). Regulation No. 392/2019, 3 May.

[14] Nagaraju S.P., Shenoy S.V., Gupta A. (2022). Frailty in End Stage Renal Disease: Current Perspectives. Nefrología.

[15] Gute L., Zimbudzi E. (2023). Interventions to Reduce Falls among Dialysis Patients: A Systematic Review. BMC Nephrol..

[16] Carvalho T.C., Dini A.P. (2020). Risk of Falls in People with Chronic Kidney Disease and Related Factors. Rev. Lat. Am. Enferm..

[17] Perez-Gurbindo I., Álvarez-Méndez A.M., Pérez-García R., Cobo P.A., Carrere M.T.A. (2021). Factors Associated with Falls in Hemodialysis Patients: A Case-Control Study. Rev. Lat. Am. Enferm..

[18] Silva A.J., Frazão J., Pimenta R. (2023). Quality of Life in People with Chronic Kidney Disease Undergoing a Regular Hemodialysis Program. Rev. Enferm. Ref..

[19] Melo D.B., Ferreira F.O., Lima J.L.F., Mazetto M., de Assis Andrade P., Martins R.H.C., Barbosa D.A. (2022). Quality of Life in Patients Undergoing Hemodialysis: Integrative Review. Res. Soc. Dev..

[20] Hosseini A., Fotokian Z., Jannat Alipoor Z. (2023). The Relationship between Fear of Falling and Frailty in Older Adults Undergoing Hemodialysis. Nurs. Midwifery Stud..

[21] King S.J., Reid N., Brown S.J., Brodie L.J., Sia A.D.H., Chatfield M.D., Francis R.S., Peel N.M., Gordon E.H., Hubbard R.E. (2023). A Prospective, Observational Study of Frailty, Quality of Life and Dialysis in Older People with Advanced Chronic Kidney Disease. BMC Geriatr..

[22] Ministério da Saúde (2015). Regulation No. 350/2015, 22 June.

[23] Casado S.A.C., Mendes M.E.R., Preto L.S.R., Vila-Chã C.J.F., Novo A.F.M.P. (2025). Effect of Inspiratory Muscle Training in Hemodialysis Patients: A Systematic Review of Randomized Controlled Trials. Disabil. Rehabil..

[24] Silva Almeida K., Costa N.S., Lima B.N., Dos Reis Neves J.L., Pereira Barreto L.G., de Jesus Freitas L., Silva M.M., Maneschy M.d.S., Novo A.F.M.P., Chiavegato L.D. (2025). Sit-to-Stand Test to Assess Muscle Strength after Intradialytic Exercises in Chronic Kidney Disease Patients: A Systematic Review with Meta-Analysis. J. Bodyw. Mov. Ther..

[25] Wilkinson T.J., Tarca B., Lightfoot C.J., Viana J.L., Wilund K.R., Ribeiro H.S., Greenwood S., Sakkas G.K., Kistler B.M. (2025). Prescribing Physical Activity and Exercise for People with CKD: A Practical Guide by the Global Renal Exercise Network. Clin. J. Am. Soc. Nephrol..

[26] von Elm E., Altman D.G., Egger M., Pocock S.J., Gøtzsche P.C., Vandenbroucke J.P., STROBE Initiative (2007). The Strengthening the Reporting of Observational Studies in Epidemiology (STROBE) statement: Guidelines for reporting observational studies. Lancet.

[27] Coelho T.F.M. (2014). Modelo Integral de Fragilidade do Idoso (Do Constructo à Avaliação–Tilburg Frailty Indicator).

[28] Ferreira P.L., Anes E. (2010). Medição da qualidade de vida de insuficientes renais crónicos: Criação da versão portuguesa do KDQOL-SF. Rev. Port. Saúde Pública.

[29] Kimura H., Kalantar-Zadeh K., Rhee C.M., Streja E., Sy J.J. (2021). Polypharmacy and Frailty among Hemodialysis Patients. Nephron.

[30] Guerrero-Carreño S., Elías-Sanz E., Gómez-Umbert M., Quintela-Martínez M., Gabarró-Taulé T., Arias-Guillén M. (2023). Valoración de la Fragilidad en un Centro de Diálisis: ¿Son Más Frágiles los Pacientes con Diabetes?. Enferm. Nefrol..

[31] Jesus L.A., Valle Pinheiro B., Lucinda L.M.F., Rodrigues de Oliveira G.B.G., Prata Dabian Haddad M.F., Raso Vidigal A.B., Maciel J.M., Watanabe L.D., Oliveira C.C., Reboredo M.M. (2023). Factors Associated with Postural Balance in Patients with End-Stage Renal Disease on Hemodialysis. Clin. Biomech..

[32] Machfer A., Fekih N., Ammar A., Hassen H.B.H., Daab W., Amor H.H., Bouzid M.A., Chtourou H. (2025). Effects of Neuromuscular Electrical Stimulation during Hemodialysis on Muscle Strength, Functional Capacity and Postural Balance in Patients with End-Stage Renal Disease: A Randomized Controlled Trial. BMC Nephrol..

[33] Young H.M.L., Ruddock N., Harrison M., Goodliffe S., Lightfoot C.J., Mayes J., Nixon A.C., Greenwood S.A., Conroy S., Singh S.J. (2022). The Impact of Falls: A Qualitative Study of the Experiences of People Receiving Haemodialysis. Int. J. Environ. Res. Public Health.

[34] Hendra H., Sridharan S., Farrington K., Davenport A. (2022). Characteristics of Frailty in Haemodialysis Patients. Gerontol. Geriatr. Med..

[35] Nair D., Liu C.K., Raslan R., McAdams-DeMarco M., Hall R.K. (2025). Frailty in Kidney Disease: A Comprehensive Review to Advance Its Clinical and Research Applications. Am. J. Kidney Dis..

[36] Kennard A.L., Glasgow N.J., Rainsford S.E., Talaulikar G.S. (2024). Narrative Review: Clinical Implications and Assessment of Frailty in Patients with Advanced CKD. Kidney Int. Rep..

[37] Zanotto T., Mercer T.H., Linden M.L.V., Traynor J.P., Doyle A., Chalmers K., Allan N., Shilliday I., Koufaki P. (2020). Association of Postural Balance and Falls in Adult Patients Receiving Haemodialysis: A Prospective Cohort Study. Gait Posture.

[38] Yang Z.C., Lin H., Jiang G.H., Chu Y.H., Gao J.H., Tong Z.J., Wang Z.H. (2023). Frailty Is a Risk Factor for Falls in Older Adults: A Systematic Review and Meta-Analysis. J. Nutr. Health Aging.

[39] Liu X., Chen S., Liu C., Dang X., Wei M., Xin X., Gao J. (2023). Novel Risk-Factor Analysis and Risk-Evaluation Model of Falls in Patients Receiving Maintenance Hemodialysis. Ren. Fail..

[40] Shirai N., Inoue T., Ogawa M., Okamura M., Morishita S., Suguru Y., Tsubaki A. (2022). Relationship between Nutrition-Related Problems and Falls in Hemodialysis Patients: A Narrative Review. Nutrients.

[41] Clyne N., Anding-Rost K. (2021). Exercise Training in Chronic Kidney Disease: Effects, Expectations and Adherence. Clin. Kidney J..

[42] American Association of Kidney Patients (2022). How to Reduce the Risk of Falling.

[43] Goto N.A., Weststrate A.C.G., Oosterlaan F.M., Verhaar M.C., Willems H.C., Emmelot-Vonk M.H., Hamaker M.E. (2020). The Association between Chronic Kidney Disease, Falls, and Fractures: A Systematic Review and Meta-Analysis. Osteoporos. Int..

[44] Niedermann K., Meichtry A., Zindel B., Ernst M., Krafft V., Mattli R., Nast I., Wieser S., Wirz M., Brunner B. (2024). Effectiveness and Cost-Effectiveness of a Single Home-Based Fall Prevention Program: A Prospective Observational Study Based on Questionnaires and Claims Data. BMC Geriatr..

